# Supplementary material for: Molecular Dynamics Simulations of a Catalytic Multivalent Peptide–Nanoparticle Complex
Source: Int J Mol Sci. 2021 Mar 31;22(7):3624. doi: 10.3390/ijms22073624 (PMC8037132; doi:10.3390/ijms22073624)
Supplement: Supplementary file 1 [file ijms-22-03624-s001.pdf]

Supplementary Materials

Article

Molecular Dynamics Simulations of a Catalytic Multivalent Peptide–Nanoparticle Complex

Sutapa Dutta [1,2], Stefano Corni [1,2,*] and Giorgia Brancolini [2,*]

[1] Dipartimento di Scienze Chimiche, Università di Padova, 35131 Padova, Italy; sutapa.dutta@unipd.it
[2] Istituto Nanoscienze, CNR-NANO S3, via G. Campi 213/A, 41125 Modena, Italy
* Correspondence: stefano.corni@unipd.it (S.C.); giorgia.brancolini@nano.cnr.it (G.B.); Tel.: +39-049-8275295 (S.C.); +39-059-2055333 (G.B.)

Details of docking calculation:

In order to make the calculation of electrostatic potential computationally less expensive, an effective charge model (ECM) is incorporated in SDA7.2.2. code[1, 2]. First, a set of partial atomic charges (net charges of the charged atom groups) are chosen to represent charge density of the solute. The OPLS parameter was used to assign atomic charges and radii to atoms. Then, the effective charges are generated via rescaling the partial atomic charges and fitted in such a way, that the electrostatic potential computed around the solute using those effective charges, surrounded by a shell with low dielectric constant and kept within a uniform solvent, replicates that of original reaction field, as obtained in case of the polarizable solvent. During the simulation the electrostatic interaction free energy between two solutes is estimated from the interaction between effective charges of a solute and the electrostatic potential grid surrounding the other. Next, the short range electrostatic desolvation free energy between two solutes is modelled as interaction between effective charge of one solute and electrostatic desolvation potential of the other one. The non-polar desolvation interaction energy between two solutes is assumed to be proportional to the solvent accessible surface area of the solute that is obstructed by the other one. This interaction also depends on the non-polar burial surface potential of the second solute surrounding the first one. In biomolecules, prevention of overlapping between the molecules is assigned through excluded volume grids that are pre-computed around the

molecule using a grid spacing of 1 Å. If during a simulation step, one molecule penetrates the exclusion volume of the other molecule, then the move is regenerated with a different random number until it omits the overlapping.

The empirical scaling factor for weighing between long range electrostatic interaction energy and short range electrostatic desolvation term is set as 1.67 for our calculation. For non-polar desolvation, the potential depends on a scaling factor (γ) such that γ = 1.0 for all the points of the second solute, lying within a minimum distance (a =3.10 Å) from the surface of the first one, at this limit the surface of the first solute is completely occluded. Similarly, γ = 0.0 if the point is further than a maximum distance (b = 4.35 Å) from the surface of the first solute, where the presence of the second solute does not affect solvation of the first one. Finally, the factor is linearly interpolated if the distance lies in between these two limits. We use γ = 0.5 in our calculation as per observations obtained from trial and error method, to get better estimation of the potential. The surface of solute 1 that is excluded to solute 2 is computed by increasing radii of the atoms by 1.77 Å for proteins of solute 1, which have solvent accessible surface area (SASA) more than the threshold. A probe of radius 1.4 Å is used as a representative of solvent (water) molecule to calculate the SASA of the solute. Spacing of the exclusion grid is considered ~ 0.5 Å to take into account the shape of the solute. The entire potential grid is multiplied by suitable factors to include the ionic contribution in potential grid.

Analysis protocol:

RMSD is being calculated using the following formula, $\mathrm{RMSD}(t) = \sqrt{\sum_{i=1}^{N} \frac{1}{N} |\boldsymbol{r_i(t)} - \boldsymbol{r_i(0)}\ |^2}$, after aligning all the simulated structures of the molecule with respect to its initial conformation using script in VMD[3]. Here $\boldsymbol{r_i(t)}$ is the position of ith atom at time t, and $\boldsymbol{r_i(0)}$ is that for initial configuration. N is total number of atoms of the polymer.

Here, we address radius of gyration (R_g^{pep}) through standard gmx gyrate command of GROMACS[4, 5] for this analysis. The calculation is manifested as per the formula, $R_g^{pep}(t) = \sqrt{\sum_{i=1}^{N}|\boldsymbol{r_i}(\boldsymbol{t}) - \boldsymbol{r_{com}}(\boldsymbol{t})|^2/\sum_{i=1}^{N} m_i}$, m_i is mass of ith atom and $\boldsymbol{r_{com}}(\boldsymbol{t})$ is coordinate of center of mass at time t and $\boldsymbol{r_{com}}(\boldsymbol{t}) = \sum_{i=1}^{N} m_i \boldsymbol{r_i}(\boldsymbol{t})/\sum_{i=1}^{N} m_i$.

We use standard g_sasa command of GROMACS[4, 5] to generate SASA of system of interest ($SASA_{sys}$) and the corresponding histogram ($H(SASA_{sys})$) over simulated data. A solvent representative sphere of radius r_{solv} is rolled over the envelop of the van der Waals surface of biotin, where this surface is composed of interlocking spheres of appropriate van der Waals radius consistent to the atom. Thus, SASA of an atom of radius r is defined as area of sphere with radius $R = r + r_{solv}$, given the fact the solvent sphere is in contact with the respective atom without penetrating any other atoms.

We also use radial distribution function, the probability distribution of finding a neighbour atom around a central atom within a spherical shell of radii r and $r + \Delta r$ through standard gmx rdf command.

For cluster analysis, we use standard gmx cluster command with gromos algorithm of GROMACS[4, 5]. This counts the number of neighbours using a threshold value of RMS ~ 0.1 nm of the structures with respect to a reference structure. The structure with largest number of neighbours with all its neighbours is considered as one cluster and after eliminating this particular cluster, the process is repeated for other structures of the trajectories. The structure with the smallest average distance to the others or the average structure or all structures for each cluster will be written to a trajectory file.

Mean squared displacement (MSD) of the center of mass of peptide over simulated trajectory is computed using g_msd command and by the algorithm as[6], $< r^2(t) >= \frac{1}{N}\sum_{i=1}^{N} < |\boldsymbol{r_i}(\boldsymbol{t}) - \boldsymbol{r_i}(\boldsymbol{0})|^2 >$. Here, third bracket denotes ensemble average, $\boldsymbol{r_i}(\boldsymbol{t})$ denotes the coordinates of the ith atom of a given time interval t starting from initial position, $\boldsymbol{r_i}(\boldsymbol{0})$. N is total number of atoms of atoms. The diffusion

coefficient D is obtained from slope of the equation a $< r^2(t) >= 6Dt$ at long time limit. Whereas coordinate for center of mass is $\boldsymbol{r_{COM}(t)} = \sum_{i=1}^{N} \frac{m_i \, \boldsymbol{r_{i\,(t)}}}{m_i}$, m_i is the mass of all ith atoms.

For intra-molecular hydrogen bond analysis of imidazole group of Histidine, we use HBonds tool in VMD[3], it computes occupancy of H bond formations between desired donor (D) and acceptor (A) atoms over the simulated trajectory; criteria like distance between |d-A| < 3 Å and the angle D-H-A < 20 degree are used.

References:

1. M. Martinez, N. J. Bruce, J. Romanowska, D. B. Kokh, M. Ozboyaci, X. Yu, M. A. Ozturk, S. Richter and R. C. Wade, *Journal of computational chemistry*, 2015, **36**, 1631-1645.
2. R. R. Gabdoulline and R. C. Wade, *Biophys J*, 1997, **72**, 1917-1929.
3. W. Humphrey, A. Dalke and K. Schulten, *Journal of molecular graphics*, 1996, **14**, 33-38, 27-38.
4. B. Hess, C. Kutzner, D. van der Spoel and E. Lindahl, *Journal of chemical theory and computation*, 2008, **4**, 435-447.
5. D. Van Der Spoel, E. Lindahl, B. Hess, G. Groenhof, A. E. Mark and H. J. Berendsen, *J. Comput. Chem.*, 2005, **26**, 1701-1718.
6. T. O. Skinner, S. K. Schnyder, D. G. Aarts, J. Horbach and R. P. Dullens, *Phys. Rev. Lett.*, 2013, **111**, 128301.
7. E. Vanquelef, S. Simon, G. Marquant, E. Garcia, G. Klimerak, J. C. Delepine, P. Cieplak and F. Y. Dupradeau, *Nucleic acids research*, 2011, **39**, W511-517.

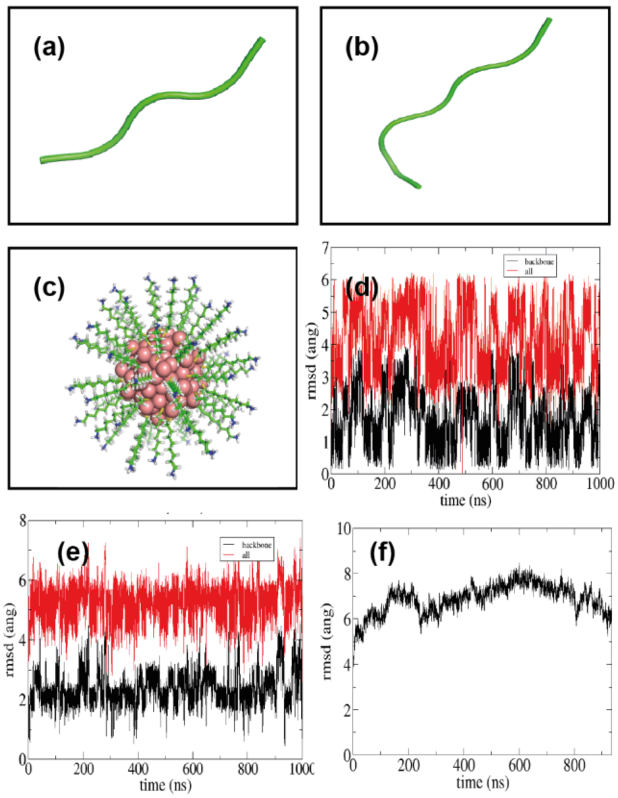

Figure S1. (a) Model structure of shorter peptide, H_1 and that for (b) longer one, H_3. (c) Model structure of Au144(L)60(L=S$(CH_2)_8NH_2^+$) as obtained from NanoModeler, gold: sphere, functionalized ligand: stick . (d) RMSD of backbone and for entire peptide (black: backbone, red: all) as function of simulation time for H_1 and (e) that is for H_3. (f) RMSD plot for ligands for simulation of Au144(L)60.

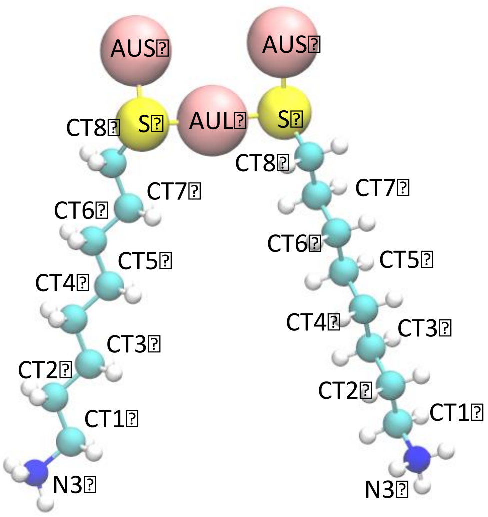

Atom	Charge
N3	-0.2607
H	0.2999
H	0.2999
H	0.2999
CT1	0.0261
HP	0.0918
HP	0.0918
CT2	-0.0278
HC2	0.0375
HC2	0.0375
CT3	-0.0202
HC3	0.0217
HC3	0.0217
CT4	-0.0088
HC4	0.0160
HC4	0.0160
CT5	0.0133
HC5	0.0115
HC5	0.0115
CT6	0.0355
HC6	-0.0041
HC6	-0.0041
CT7	0.0269
HC7	0.0028
HC7	0.0028
CT8	-0.0551
H8	0.0827
H8	0.0827
S	-0.1688
AUS	0.0003
AUL	0.0396

Figure S2. Partial charges of the nano-cluster are derived for the surface atoms of the smallest interfacial repeating unit, namely AUS-RS–AUL–SR-AUS. Where the SR=alkanethiol functional groups is connected to the core with a sulfur atom forming the so-called “staples” as in figure, where the sulfur atom is forming a covalent bond with one gold atom at the interface (AUL=gold ligand) and a gold atom at the surface (AUS = gold surface). Using R.E.D. server[7] the partial charges are evaluated from density functional (DFT) calculations (b3lyp/6-31G*//b3lyp/6-31G*) and Connolly surface algorithm is used in MEP computation (2 stage RESP fit qwt=0.0005/0.001).

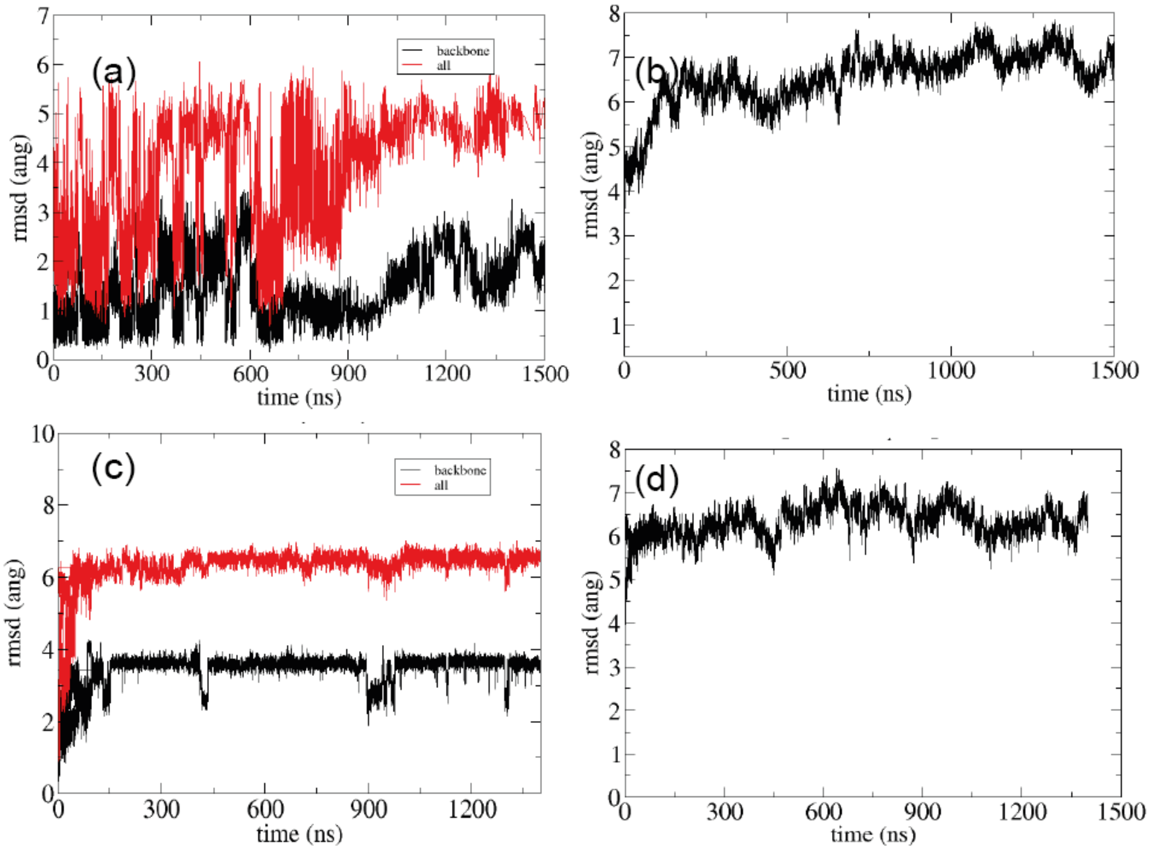

Figure S3. (a) RMSD plots for peptide (black: backbone, red: all) for H_1 bound with Au144(L)60 and (b) for the ligands in same system. (c) RMSD plots for peptide (black: backbone, red: all) for H_3 bound with Au144(L)60 and (d) for the ligands in same system as function of simulation time.

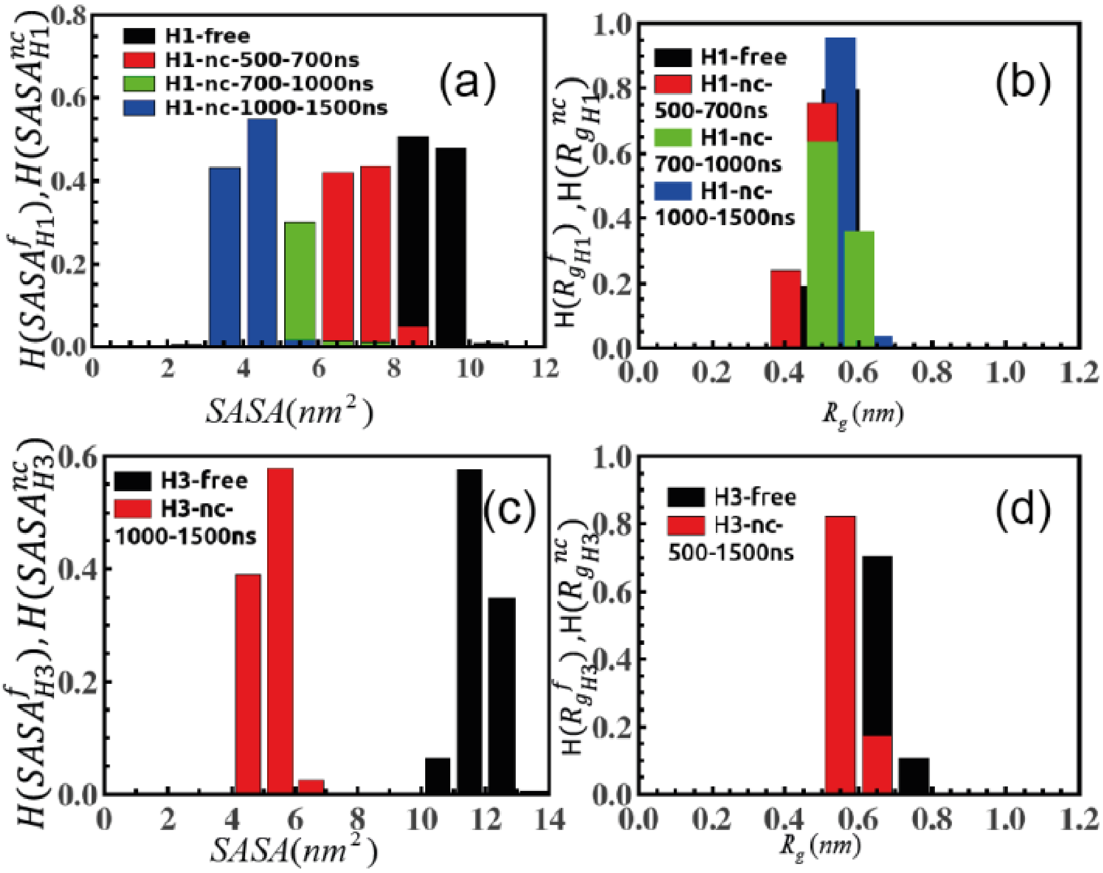

Figure S4. (a) Distribution of SASA of H_1 in free and in presence of gold-cluster. (b) Distribution of radius of gyration for the same in free state and in presence of gold-cluster. (c) Histogram of SASA for H_3 in free and with nano-cluster, that for (d) Distribution of radius of gyration for longer peptide in two different environments.

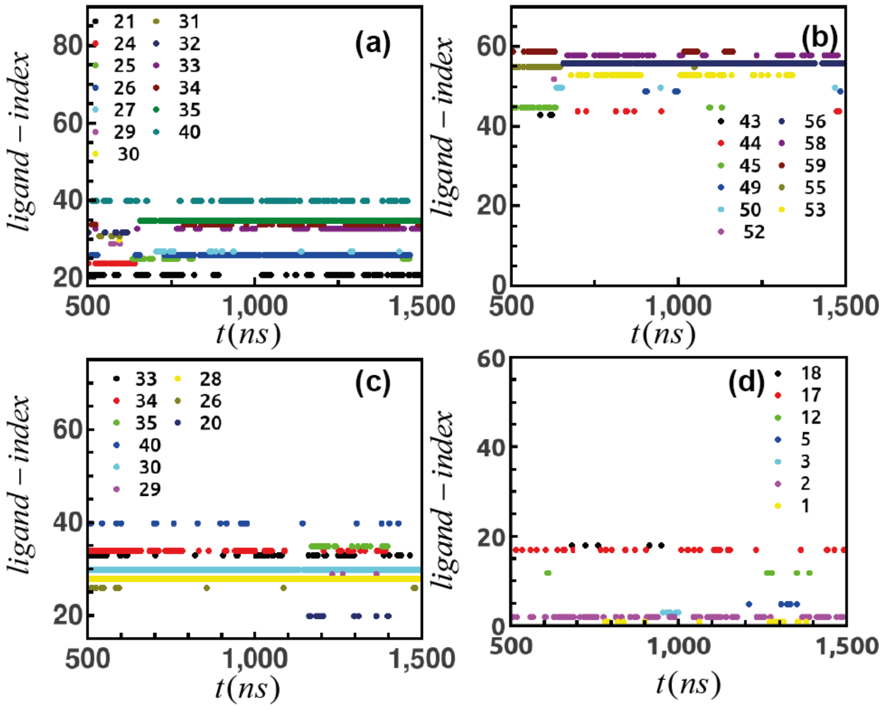

Figure S5. (a)-(b) Ligands that make contact with peptide H_1simultaneously over simulation time. (c)-(d) Ligands that make contact with peptide H_3 simultaneously over simulation time.

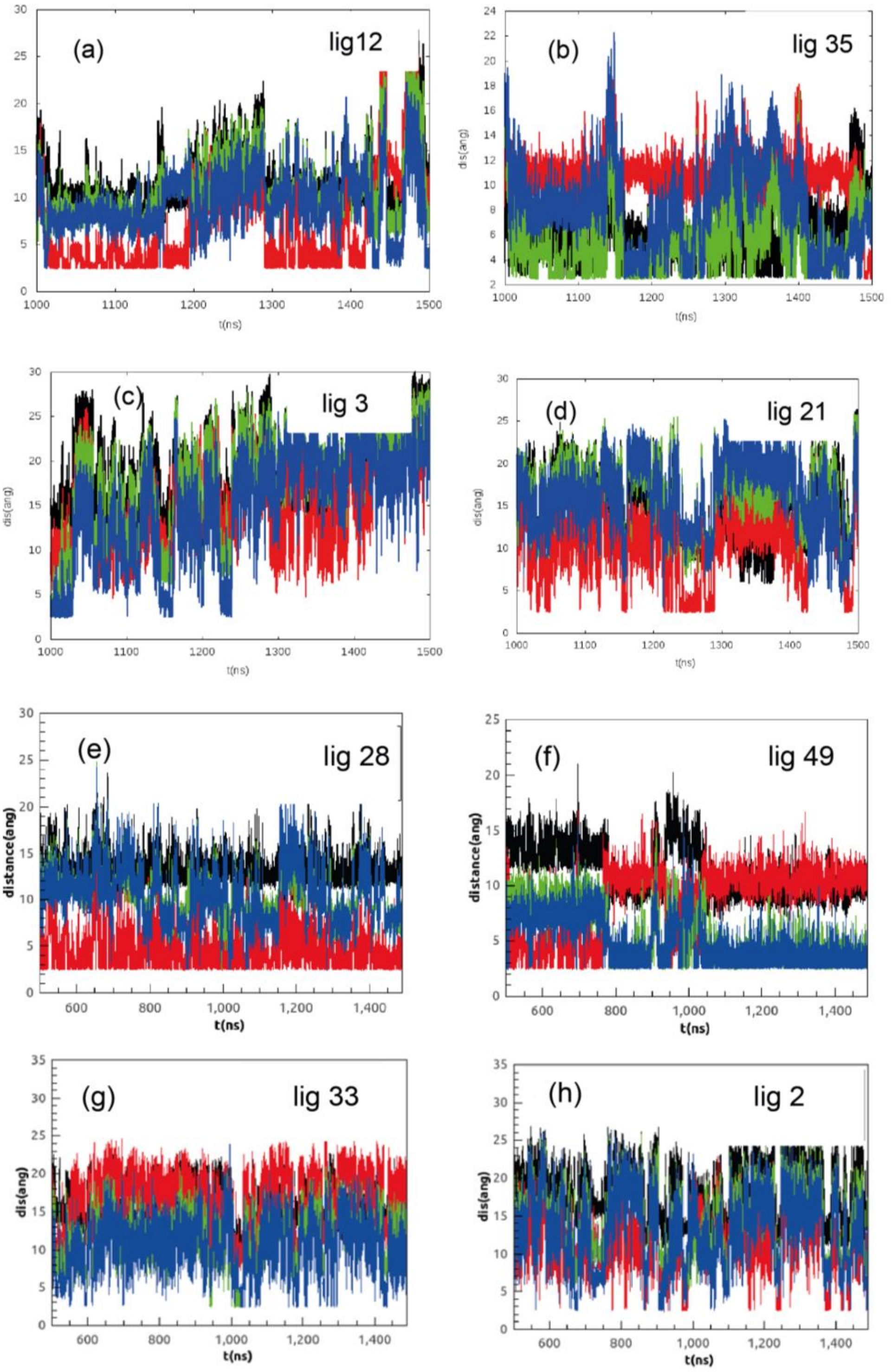

Figure S6. (a)-(b) Some representative cases for ligands making strong contact with different atoms of different residues of shorter peptide with simulation time, carbonyl O atom of 1H (black), the side chain carboxyl oxygens of 3D (red) and 4D (green), the terminal carboxyl oxygen of the 5D (blue). (c)-(d) Ligands making weak contact with same group of atoms of above mentioned group of residues of

shorter peptide with simulation time. (e)-(f) Ligands making strong contact for H_3; carbonyl O atom of 2H (black), the side chain carboxyl oxygens of 5D (red) and 6D (green), the terminal carboxyl oxygen of the 7D (blue)and that for (g)-(h) weaker contact.

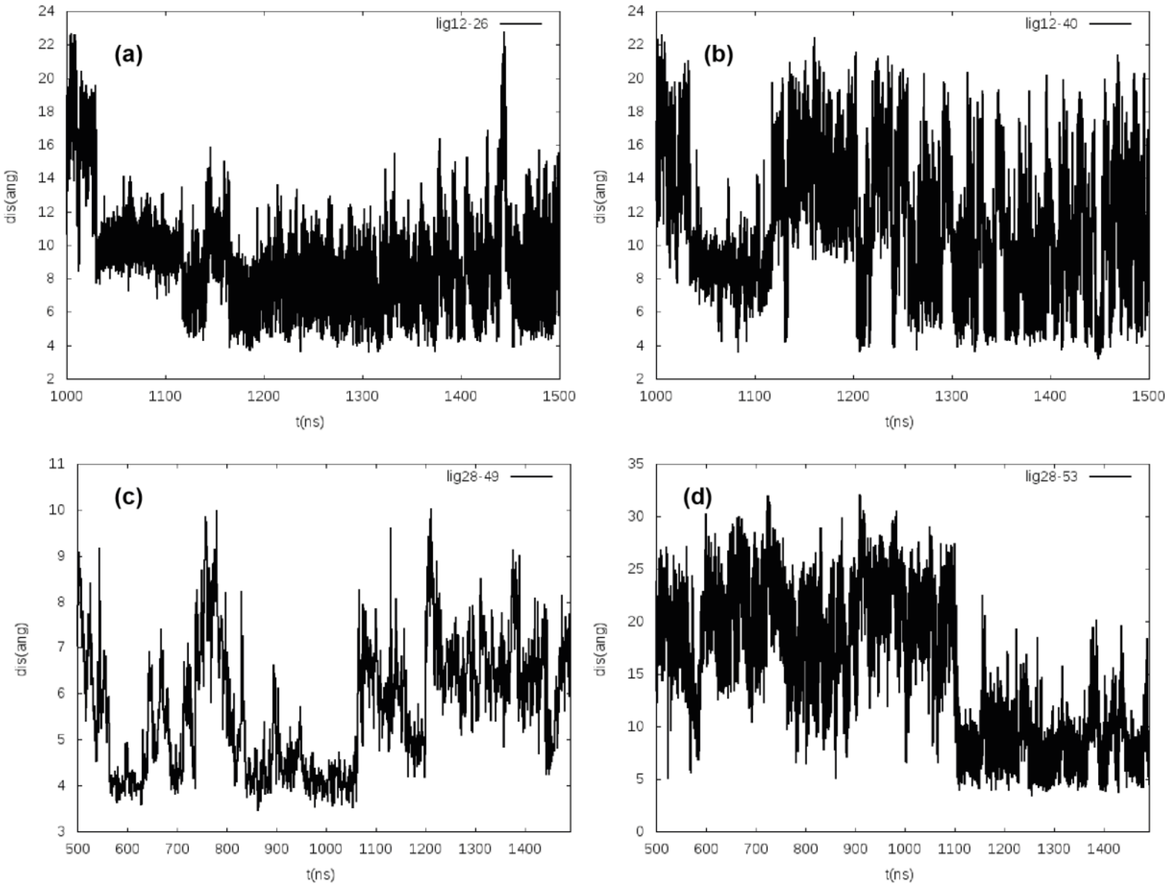

Figure S7. (a)-(b) Some representative cases for distance fluctuations between ligands making simultaneous strong contact with shorter peptide over simulation time, (c)-(d) Distance fluctuations between ligands making simultaneous strong contact with longer peptide over simulation time.

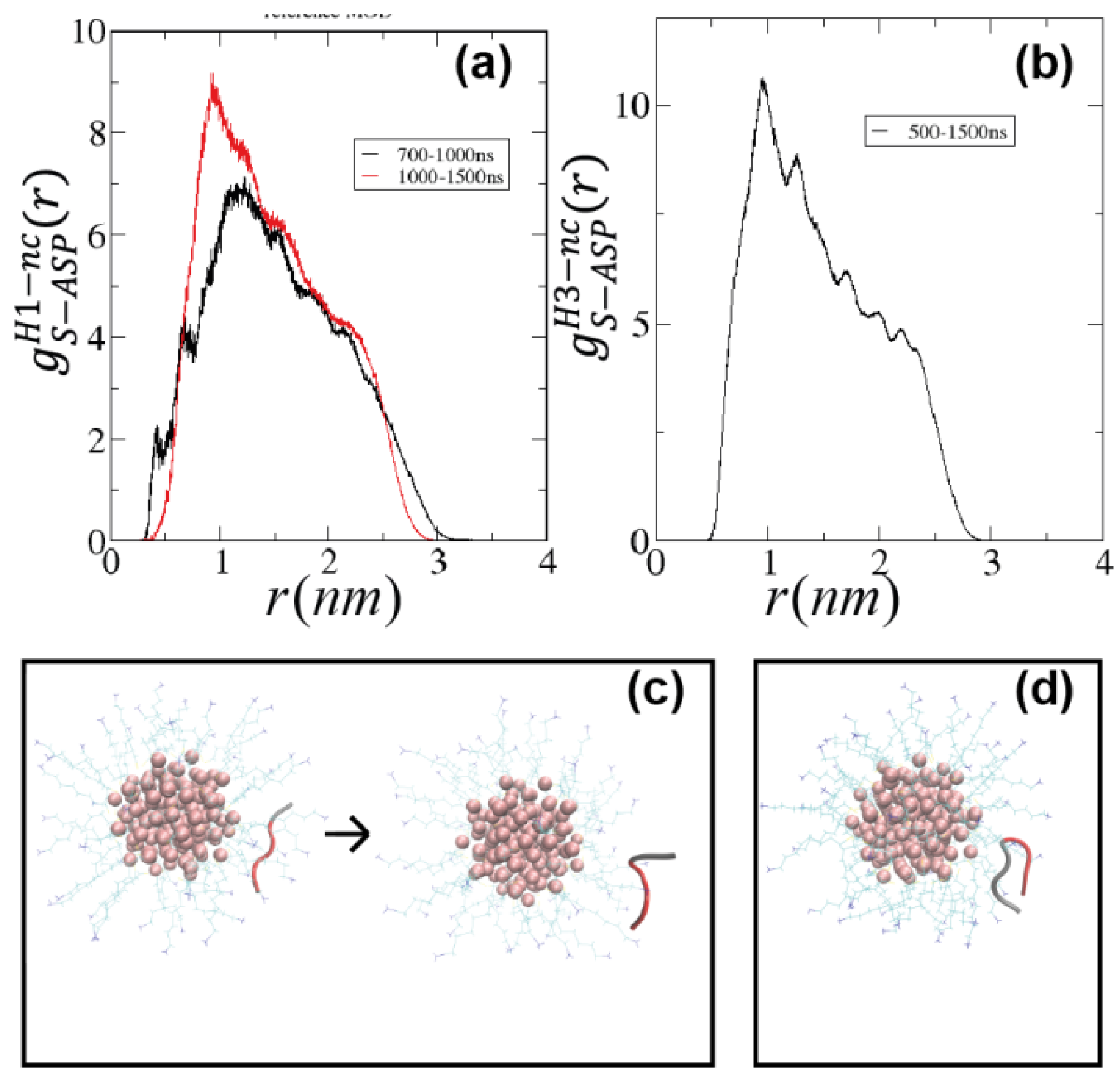

Figure S8. (a) Pair correlation for S and ASP residue of shorter peptide for two different time intervals over simulation and same for (b) longer peptide for last 1 μs in case of longer peptide in presence of gold cluster. (c) Snapshot indicating penetration of shorter peptide inside monolayer for 700-1000ns and the peptide comes back at the surface eventually. (d) Longer peptide assembles at surface of monolayer after simulation.

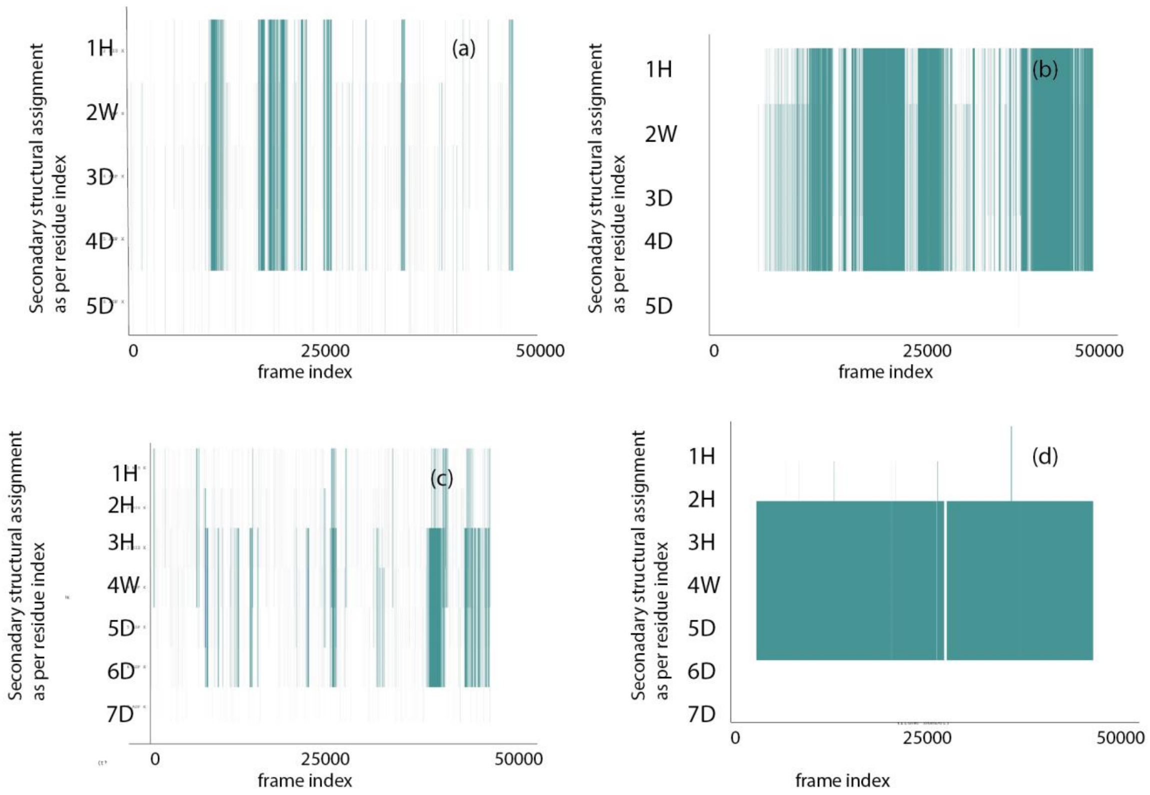

Figure S9. Secondary structural assignment generated over simulated trajectories for each residue of both peptides in free as well as in presence of nano-cluster indicating increasing in percentage of β-turn in bound form (cyan) than random coil (white part); (a) H_1^f, (b) H_1^{nc}, (c) H_3^f, (b) H_3^{nc}.

Table S1. Details of intra-molecular H bond network for Histidine for peptides in two different environments.

System	H bond index	Donor atom	Acceptor atom
H_1 free	1	HIS1-Side-NE2	ASP4-Side-OD1
H_1 free	2	HIS1-Side-NE2	ASP4-Main-O
H_1 free	3	HIS1-Side-NE2	ASP3-Side-OD1
H_1 free	4	HIS1-Side-NE2	ASP5-Side-OD1
H_1 free	5	HIS1-Side-NE2	ASP3-Side-OD2
H_1 free	6	HIS1-Side-NE2	ASP4-Side-OD2
H_1 free	7	HIS1-Side-NE2	ASP5-Side-OD2
H_1 free	8	HIS1-Side-NE2	ASP5-Side-O1
H_1 free	9	HIS1-Side-NE2	ASP5-Side-O2
H_1 nano-cluster	1	HIS1-Side-NE2	ASP3-Side-OD1
H_1 nanocluster	2	HIS1-Side-NE2	ASP3-Main-O
H_1 nanocluster	3	HIS1-Side-NE2	ASP4-Side-OD2
H_1 nanocluster	4	HIS1-Side-NE2	ASP4-Side-OD1
H_1 nanocluster	5	HIS1-Side-NE2	ASP3-Side-OD2
H_3 free	1	HIS3-Side-NE2	ASP5-Side-OD2
H_3 free	2	HIS2-Side-NE2	TRP4-Side-NE1
H_3 free	3	HIS1-Side-NE2	HIS3-Side-ND1
H_3 free	4	HIS3-Side-NE2	ASP5-Side-OD1
H_3 free	5	HIS2-Side-NE2	ASP7-Side-OD2
H_3 free	6	HIS2-Side-NE2	ASP7-Side-OD1
H_3 free	7	HIS3-Side-NE2	ASP6-Main-O
H_3 free	8	HIS3-Side-NE2	ASP6-Side-OD1
H_3 free	9	HIS2-Side-NE2	ASP7-Side-OD1
H_3 free	10	HIS2-Side-NE2	ASP7-Side-O2
H_3 free	11	HIS1-Side-NE2	ASP6-Side-OD2
H_3 free	12	HIS1-Side-NE2	ASP6-Side-OD1
H_3 free	13	HIS3-Side-NE2	ASP6-Side-OD2
H_3 free	14	HIS2-Side-NE2	ASP6-Side-OD1
H_3 free	15	HIS2-Side-NE2	ASP6-Main-O
H_3 free	16	HIS1-Side-NE2	ASP7-Side-OD1
H_3 free	17	HIS3-Side-NE2	ASP7-Side-OD1
H_3 free	18	HIS1-Side-NE2	ASP7-Side-OD2
H_3 free	19	HIS2-Side-NE2	ASP6-Side-OD2
H_3 free	20	HIS3-Side-NE2	ASP7-Side-OD2
H_3 free	21	HIS3-Side-NE2	ASP7-Side-O2
H_3 free	22	HIS3-Side-NE2	ASP7-Side-O1
H_3 free	23	HIS1-Side-NE2	ASP5-Side-OD2
H_3 free	24	HIS1-Side-NE2	ASP5-Main-O
H_3 free	25	HIS1-Side-NE2	ASP7-Side-O1
H_3 free	26	HIS1-Side-NE2	ASP6-Main-O
H_3 free	27	HIS1-Side-NE2	ASP7-Main-O2

H_3 nano-cluster	1	HIS3-Side-NE2	ASP5-Side-OD1
H_3 nano-cluster	2	HIS1-Side-NE2	ASP5-Main-O
H_3 nano-cluster	3	HIS1-Side-NE2	ASP6-Side-OD2
H_3 nano-cluster	4	HIS3-Side-NE2	ASP5-Side-OD2
H_3 nano-cluster	5	HIS1-Side-NE2	ASP6-Side-OD1
H_3 nano-cluster	6	HIS3-Side-NE2	ASP7-Side-OD1
H_3 nano-cluster	7	HIS1-Side-NE2	ASP7-Side-OD2
